# Parent and Friend Relationship Quality and Links to Trajectories of Loneliness During the First Year of College

**DOI:** 10.1007/s10578-022-01416-6

**Published:** 2022-09-24

**Authors:** Maria D. Calderon Leon, João F. Guassi Moreira, Natalie M. Saragosa-Harris, Yael H. Waizman, Anna Sedykin, Tara S. Peris, Jennifer A. Silvers

**Affiliations:** University of California, A191 Franz Hall, 502 Portola Plaza, Los Angeles, CA 90095-1563 USA

**Keywords:** Adolescence, Emerging adulthood, Loneliness, Parent relationships, Friend relationships

## Abstract

Beginning college involves changes that can increase one’s vulnerability to loneliness and associated negative outcomes. Parent and friend relationships are potential protective factors against loneliness given their positive association with adjustment. The present longitudinal study, with data collection at baseline, 1 month, and 2 months later, assessed the comparative effects of self-reported parent and friend relationship quality on loneliness in first-year college students (*N* = 101; 80 female, M_age_ = 18.36). At baseline, parent and friend relationship quality were negatively associated with loneliness. Longitudinal data revealed that friend relationship quality interacted with time, such that its effects on loneliness attenuated over the course of 2 months. By contrast, parent relationship quality continued to predict lower loneliness 2 months post-baseline. These results highlight the importance of close relationships and suggest that targeting relationship quality could be effective in helping youth transition to college.

**Supplementary Information:**

The online version contains supplementary material available at 10.1007/s10578-022-01416-6.

## Introduction

Human development features an abundance of transitions—periods in which biological, social, or cultural contexts change quickly over a short amount of time. One transition that is particularly important for adjustment over the lifespan is the transition from adolescence to adulthood [1, 2]. While decades-old questions and debates about when adolescence ends persist today [3–5], scientists generally agree that the transitional period between adolescence and adulthood is a critical yet understudied window that has important consequences for adjustment [6–9]. The importance of this window is underscored not only by the panoply of changes in interpersonal relationships, sociocultural environments, and educational/occupational expectations—but also by its endproduct: adult independence. This phase is further complicated by the onset of many forms of psychopathology, such as anxiety and depression, during this interval [10], trends that emerge during a period of decreased utilization of mental health services [11].

For many youth, a key event in the transition to adulthood is beginning college, which often involves multiple changes, including moving away from home and navigating a novel social context [12]. While rich in opportunity, these changes increase vulnerability to loneliness which in turn affects physical, academic, and psychological well-being [13–15]. Therefore, it is vital to identify protective factors against loneliness during the first year of college. Strong relationships with parents and friends are likely protective factors against loneliness given their association with psychosocial adjustment in young adults [16, 17]. However, little research has assessed the comparative effects of parent and friend relationship quality on subjective loneliness during the first year of college. Additionally, few studies have characterized normative trajectories of loneliness across the first year of college. The present study sought to fill these gaps in the literature by examining the association between parent and friend relationship quality and loneliness during the first year of college while simultaneously quantifying normative trajectories of loneliness during this period. Such work is crucial for crafting interventions aimed at supporting successful adjustment in college, and, more broadly, the transition to full independence.

Although a time for self-discovery and new experiences, entering college is also a source of stress for many [18, 19]. It is common for new college students to struggle with the discrepancy between their expectations of college and their actual experiences [20, 21]. Individuals may also struggle with an uneasy sense of being caught in developmental limbo between adolescence and adulthood, by still being reliant on their parents for some aspects of life while being independent in other domains [1]. Some individual struggle with leaving home given the lack of structure, new responsibilities, and increased self discipline needed which can make the college transition more challenging [22]. Having parental support while still having the freedom to gain independence is pivotal for a successful transition. For many, their first year of college is the first time living away from their parents and simultaneously involves making new friends in college while maintaining relationships with friends back home. For these reasons and more, the first year of college, particularly the initial transition, is frequently characterized by normative decreases in psychosocial well-being and increases in psychological distress [23]. What is comparatively less studied is how this distress persists across the first year of college and what factors might exacerbate or help attenuate this distress.

Collectively, the changes that occur during the first year of college can increase one’s vulnerability to loneliness [24, 25]. Loneliness is defined here as the distressing feeling that results from the perception that one's social needs are not being met or a discrepancy between perceived quality and desired quality of relationships [26, 27]. Loneliness is not confined to any particular developmental period but it is particularly prominent in young adulthood, perhaps especially for first-year college students [28, 29]. Loneliness peaks during the first year of college relative to later years of college [24, 30, 31], particularly for individuals who move away for college [25]. While some degree of loneliness is normative during the first year of college [32], elevated levels of loneliness have been linked to cardiovascular health risk, elevated cortisol levels, diminished academic achievement, worse sleep quality, daytime dysfunction, and elevated depressive symptomatology in young adults [17, 33–36]. Further evidence also suggests that loneliness predicts college drop-out, over and above college GPA, and appears to indirectly influence use of campus counseling resources via elevated levels of depression and anxiety [15, 37]. For recent first-year college students, the COVID-19 pandemic has played an additional role in potentially increasing risk of stress and loneliness due to mandatory social distancing policies and disruptions in the ability to establish or maintain social networks [38, 39].

Given its various negative outcomes, it is imperative to identify buffers against loneliness during the first year of college. Relationships with parents and friends are potential buffers against loneliness given their association with psychosocial adjustment. Having high-quality relationships with parents and friends helps fulfill the socioemotional needs of young adults [25, 40], which in turn lessens their vulnerability for loneliness [41, 42]. Because of the differing roles of parents and friends in the lives of young adults there is no definitive answer in regards to which relationship matters more as a buffer against loneliness. Parental relationships improve and stabilize during young adulthood and there is a stronger connection to family during this time compared to earlier adolescence [16, 43]. Relationships with parents improve normatively over the first year of college [12, 16, 44, 45], and high parent–child relationship quality during this period has been linked to better academic, psychological and social well-being in college [16, 44, 45].

Friends can provide a complementary source of social support from that of parents during the first year of college [42], in part because young adults are more inclined to confide in and seek day-to-day emotional support from their friends than parents [46–48]. Friends may be particularly important in college for helping individuals achieve their social and academic goals while simultaneously establishing a deeper sense of belongingness to their university [16, 49]. Given that individuals’ friendship networks may change dramatically during the first few months of college, it stands to reason that friendships established prior to college are initially helpful at buffering against loneliness whereas those established after the onset of college may have greater impacts on loneliness over time [40]. Friendships established prior to college decrease in size and quality during the first year of college [12, 50] which can leave young adults without support if they do not develop new friendships or have strong family support networks. Importantly, friend relationship quality, as opposed to the number of friends, is a better predictor of loneliness and thus the focus of the present study was on friendship quality within one’s overall friend group rather than social network size [32].

While evidence suggests that both parent and friendship relationship quality can buffer against loneliness during the first year of college, little research has compared the relative impacts of parent and friend relationship quality, in the same sample, on well-being during the first year of college as well as whether their effects are additive or interactive. Moreover, few prior studies have examined loneliness as an outcome measure in college students, instead using it as a predictor of adjustment outcomes such as academic performance, retention, and mental health [15, 51, 52]. Further, studies that examine loneliness often assess it at only one or two timepoints rather than characterizing longer-term trajectories (e.g., [17]). The present study sought to fill these knowledge gaps by first characterizing normative changes in loneliness over the first year of college, and second, to test how loneliness during the first year of college varies depending on parent and friend relationship quality. Specifically, we were interested in determining the associations between parent and friend relationship quality and loneliness in addition to whether one type of relationship was more strongly associated with loneliness outcomes. This feature of our study is noteworthy because most prior studies focus on relationship quality with close others as a whole, or with only one particular type of social other (solely friends, or solely parents) [17]. Studying the unique contributions of both parents and friends has the added benefit of helping identify eventual mechanistic specificity. Furthermore, given that most prior work on loneliness during the first year of college has focused on the effect of loneliness on other adjustment outcomes or has assessed loneliness at only one or two timepoints (e.g., [17]), we sought to quantify normative longitudinal trajectories of loneliness for the first time. Characterizing loneliness trajectories could help identify trends useful for targeting loneliness during college and provide overall insight into individuals’ experiences of loneliness. Identifying normative loneliness trends during the first year of college could also provide essential context to understand how fluctuations in loneliness impact the overall college experience. These study aims resulted in four a priori hypotheses, as well as an exploratory, post hoc (i.e., following data collection) research question. Finally, the COVID-19 pandemic required us to consider not only how individuals adapted to college but also to a second major stressor—a global pandemic. At the time of the study, it was unknown how COVID-19 could impact the already challenging transition to college. For this reason, we conducted exploratory analyses to examine the impact of the pandemic on loneliness over time.

We had the following hypotheses:

1. Given the role of parent and friend relationships in psychosocial adjustment, we hypothesized negative associations between parent relationship quality and loneliness as well as friend relationship quality and loneliness at baseline.
2. We hypothesized there would be a difference in the magnitude of the negative association between relationship quality and baseline loneliness for parents compared to friends given their differing influences on young adults. Because prior work has independently linked both parent and friend relationship quality to well-being in young adults, this hypothesis was non-directional.
3. We hypothesized that baseline loneliness would be lowest for individuals with both high parent and high friend relationship quality. In other words, parent and friend relationship quality would interact with each other to predict lower levels of loneliness [53].
4. We hypothesized that high relationship quality with parents and friends at baseline would predict less loneliness 1 month and 2 months post-baseline.

Exploratory research question:

1. What are typical trajectories of loneliness across the first year of college? We aimed to characterize loneliness trajectories and contextualize the longitudinal effects of parent and friend relationship quality on loneliness.

## Methods

### Participants

The sample consisted of 101 (M_age_ = 18.36, SD = 0.48, range 18–19; 80 female) first-year college students from a large, public university in the Western United States. Participants reported their race as 37% Asian, 27% Caucasian, 10% African American, 2% Native American Indian or Alaska Native, 12% multiracial, 8% other, and 4% declined to report. Additionally, 24% of participants self-identified as Hispanic or Latinx.

### Measures

#### Parent and Friend Relationship Quality

Parent and friend relationship quality were assessed at baseline with the Inventory of Parent and Peer Attachment Mother, Father, and Peer versions (IPPA; [51]). The measure consists of 25 items across three subscales: trust (e.g. “My mother respects my feelings”), communication (e.g. “When we discuss things, my friends care about my point of view”), and alienation (e.g. “I get upset easily around my father”). Participants used a five-point Likert scale to rate the frequency of each item (1 = “Almost never or never” to 5 = “Almost always or always”). Negatively worded items were reverse-scored when calculating relationship quality scores. Prior work has extensively used the IPPA as a measure of relationship quality [54, 55]. Participants completed the IPPA Mother version to assess their relationship with their mother and responses were averaged into a mean mother relationship quality score (Mother IPPA: ω = 0.96). Likewise, the IPPA Father version was completed, if applicable, and responses were averaged into a mean relationship quality score for father (Father IPPA: ω = 0.96). Mean mother and father relationship quality scores (*r* (89) = 0.52, p < 0.01) were then averaged to yield one parent relationship quality score. Greater scores on the measure indicated stronger parent relationship quality. In the case of participants who only reported relationship quality for one parent (N = 12), responses for the lone parent were used as their final measure of relationship quality. The IPPA Peer version was utilized as a measure of overall, general relationship quality with their group of friends and responses to the measure subscales were averaged [56] to yield one friend relationship quality score that represented overall relationship quality with their friend group (Peer IPPA: ω = 0.96). Any missing responses to items of the IPPA Mother, Father, or Peer were excluded from calculations of mean relationship quality scores.

We also collected a single item measure asking participants to describe the extent to which their interactions with friends were more comprised of those with older (presumably those prior to college), compared to newer (presumably those made at college), friends (“I rely on my old friends more than my new ones”). Participants rated their agreement (“yes”) or disagreement (“no”) with the item. This item was collected to help unpack any potential moderating effects of relying on friends established prior to college (or, alternately, relying on new friends) on the relationship between friend relationship quality and loneliness.

#### Loneliness

Subjective loneliness was assessed at baseline, 1 month, and 2 months later with the UCLA Loneliness Scale (Version 3; [57]). The UCLA Loneliness Scale consists of 20 items, such as “How often do you feel there is no one you can turn to?” and “How often do you feel left out?”*.* Participants indicated their responses on a four-point Likert scale (1 = “Never” to 4 = “Always”). Positively worded items were reverse-scored and responses were averaged to yield one loneliness score such that higher scores indicated greater levels of loneliness. Any missing responses to items of the loneliness scale were excluded from average loneliness score calculations. Critically, scores from our data evinced good reliability (time 1: ω = 0.95, time 2: ω = 0.95, time 3: ω = 0.94).

### Procedure

First-year college students were contacted during their first quarter of college via their university emails with an invitation to participate in the study. Participants were eligible if they were 18–25 years old, were first-year college students (i.e., never previously attended an undergraduate institution), spoke English fluently, and did not have any serious medical or psychiatric conditions. Individuals who expressed interest and qualified following a brief email screening were scheduled for an in-person lab session during their Fall or Winter quarter of college, depending on availability. Upon arriving at the lab session, participants completed informed consent. The lab session consisted of completing various self-report measures and computerized assessments. All measures that were used for this study are described below; unanalyzed measures collected are disclosed in the Supplement. Similarly, all analyses conducted for this study are disclosed here or in the Supplement. Participants were compensated $20 (USD) for their participation in the baseline session.

**Fig. 1:**
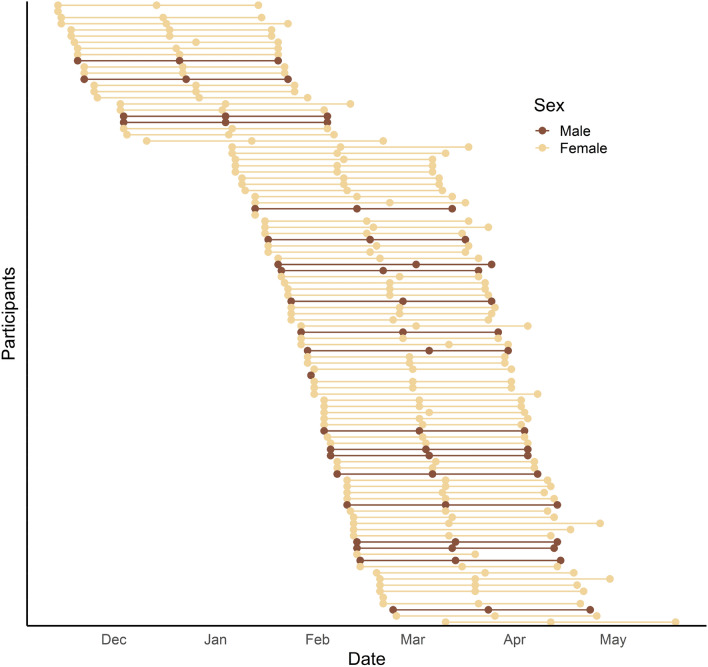
Visualization of data collection over time

Following the laboratory visit, participants completed two online follow-up assessments during the same academic year, consisting of self-reported loneliness (along with other orthogonal measures), 1 month and 2 months after baseline. Data collection for this report took place between November 14th, 2019 (approximately 5 weeks into the fall quarter) and May 20th, 2020 (approximately half-way through the spring quarter). The initial study was slated to end after the third timepoint, but additional waves of data collection occurred beyond the academic year for the purpose of collecting data specifically about the COVID-19 pandemic. These data were not analyzed here due to extending beyond the focus of the current research questions. Attrition over the study was minimal—94 participants provided complete data for all three time points, 3 participants provided data for two time points, and only 4 participants provided data for only one time point, totaling 292 available data points for longitudinal analysis. All participants provided data for the first timepoint. Figure 1 visualizes the timing of data collection for each participant. Participants were compensated $25 for participating in the longitudinal portion of the study. The local Institutional Review Board approved all procedures and methods. Data, materials, and analysis code for this study are publicly available on the Open Science Framework (osf.io/4x6ef).

### Data Analysis

Analyses were completed in two stages: baseline and longitudinal. Baseline analyses allowed us to capture a snapshot of the contemporaneous associations between relationship quality and loneliness at the beginning of the study, whereas longitudinal analyses allowed us to track within-person changes in trajectories of loneliness over time. All analyses were conducted using R, the statistical software package [58].

### Baseline

We conducted Pearson’s correlation analyses using the *psych* package [59] to examine the correlation between parent and friend relationship quality and loneliness as well as between parent and friend relationship quality. We then ran two ordinary least squares multiple linear regression analyses using the lm() function to predict loneliness (dependent variable). First, we assessed independent associations between parent and friend relationship quality (controlling for sex; dummy coded female = 0, male = 1) and loneliness (hypothesis 1). In the second model, we added the interaction term for parent and friend relationship quality (hypothesis 3) to the main effects of parent and friend relationship quality, while controlling for sex. Since baseline age was bound between 18 and 19 years, we did not include age as a covariate. We also ran a post-hoc multiple linear regression analysis with an interaction term for friend relationship quality and the single-item question about relying on older or new friends, to test if that moderated the relationship between friend relationship quality and baseline loneliness. Lastly, we computed semi-partial correlations to examine whether there was a difference in the magnitude of the effect of parent and friend relationship quality on loneliness (hypothesis 2). All baseline data were collected prior to the onset of the COVID-19 pandemic.

### Longitudinal

We conducted linear growth curve modeling analyses in a multilevel modeling framework using the *nlme* package [60] to determine whether parent and friend relationship quality (assessed at baseline) predicted loneliness outcomes longitudinally (hypothesis 4) as well as to characterize normative loneliness trajectories (exploratory research question). Growth curve analyses were chosen as the analysis method given the longitudinal nature of the data and the need to account for time-varying changes in loneliness as well as between-person effects. Our model building and evaluation procedures consisted of a stepwise procedure that involved first specifying the most parsimonious model possible given our hypotheses, and then progressively adding covariates to eliminate potential confounds and testing for higher order interaction effects between variables of interest.

Our first modelling step predicted self-reported loneliness as a function of time (coded as days since subject-specific baseline, baseline equaling zero), parent relationship quality, and friend relationship quality while allowing the effect of time and the intercept to vary randomly across participants. Notably, the centering of our time variable means that coefficients associated with time will not reflect between-subject differences in average loneliness scores [61]. The second step included all the aforementioned effects while statistically adjusting for sex (dummy coded female = 0, male = 1), the academic quarter the participant enrolled in the study (dummy coded fall = 0, winter = 1), and whether any of a given participant’s datapoints were collected during the COVID-19 pandemic (dummy coded all datapoints collected before COVID-19 pandemic = 0, at least one datapoint collected during COVID-19 pandemic = 1) (all time invariant, between-subject predictors). Given our sample, we defined this variable using the day the university cancelled in-person instruction (March 13th, 2020). The third and final step added interaction terms between time and the relationship quality variables (time × parent relationship quality; time × friend relationship quality). Parent and friend relationship quality were centered at their respective means before modeling. Additionally, because standard multilevel modeling assumptions of uncorrelated residuals are typically unrealistic in the context of growth curve modeling [62], the level 1 error variance/covariance structure was modeled with an imposed first order autoregressive structure (AR(1)). This structure assumes that the covariance in residual within-person errors between two time points with lag = 1 is greater than a pair of time points with lag = 2. The multiple equations form of the final model (i.e., that with all predictors included) is notated below and Table 3 contains statistical output for all models.

Within-person (level 1):

$$Loneliness_{ti} = \pi_{0i} + \pi_{1i} Time_{ti} + e_{ti} .$$

Between-person (level 2):

$$\begin{aligned} \pi_{0i} & = \gamma_{00} + \gamma_{01} ParentRQ_{i} + \gamma_{02} FriendRQ_{i} + \gamma_{03} Sex_{i} + \gamma_{04} Quarter - Start_{i} + \gamma_{05} Any - COVID_{i} + u_{0i} \\ \pi_{1i} & = \gamma_{10} + \gamma_{11} ParentRQ_{i} + \gamma_{12} FriendRQ_{i} + u_{1i} . \\ \end{aligned}$$

*Loneliness*_*ti*_ refers to the self-reported loneliness scores on the *t-*th day since baseline for the *i*-th individual. The first between-person equation represents the main/simple effects of between-person predictors, whereas the second between-person equation summarizes interactions with time (i.e., longitudinal trajectories that change as a function of between-person variables collected at baseline). The fixed-effect intercept ${\gamma }_{00}$ represents the grand mean loneliness score at baseline for a male participant who began the study in the fall quarter, did not have any follow-up data points collected during the COVID-19 pandemic, and reported average parent and friend relationship quality. The γ_10_ coefficient represents the effect of time (in days since baseline) on loneliness, conditional on mean levels of parent and friend relationship quality. The γ_01_ and γ_02_ coefficients represent the effects of parent relationship quality and friend relationship quality on baseline loneliness (e.g., conditional on time equal to zero); the γ_03_–γ_05_ coefficients represent the effects of sex, starting quarter, and the COVID-19 pandemic on loneliness. The γ_11_ and γ_12_ coefficients respectively represent interactions between time and parent relationship quality, and time and friend relationship quality. In other words, these two coefficients can be interpreted as differences in loneliness trajectories given 1 unit differences in parent and friend relationship quality at baseline. The ${u}_{0i}$ term represents the stochastic component of random intercepts, conditional upon parent and friend relationship quality and aforementioned covariates (i.e., a subject-specific deviation from the conditional intercept). The ${u}_{1i}$ term represents the stochastic component of random time slopes, conditional upon parent and friend relationship quality (i.e., a subject-specific deviation from a conditional slope of time). Lastly,${e}_{ti}$ denotes within-subject error when predicting loneliness from time, between-person predictors, and interaction terms.^1^

We ran additional, supplemental models containing expanded demographic variables that included race, ethnicity, and first-generation status. Given that no direct measure of socioeconomic status was obtained, first-generation status was utilized as a rough estimate of socioeconomic status. Because not all participants provided data on race and thus reduced the sample size for these analyses (4 participants, resulting in the loss of 9 longitudinal observations), we only feature models here with variables that had complete data. Notably, the key findings involving time, relationship quality, and their interaction are the same between the models reported here and the supplemental models with additional demographic variables. Findings involving these additional demographics are briefly noted below and reported in greater detail in the Supplement.

## Results

### Descriptive Statistics

Means and standard deviations for all study variables are listed in Table 1 and bivariate correlations are presented in Table 2. Loneliness at time 1, time 2, and time 3 were positively correlated with each other. Loneliness was negatively correlated with parent and friend relationship quality at all three time points. Additionally, parent relationship quality was positively correlated with friend relationship quality.

**Table 1:** Means and standard deviations for study variables

Variables	N	M (SD)
T1 loneliness	101	2.18 (0.57)
Parent RQ	101	3.53 (0.77)
Friend RQ	101	4.12 (0.63)
T2 Loneliness	95	2.25 (0.55)
T3 Loneliness	96	2.23 (0.54)

Variables	N	%
Sex		
Females	80	79.2
Males	21	20.8
Starting quarter		
Fall	23	22.8
Winter	78	77.2
Any data during COVID-19?		
Yes	68	67.3
No	33	32.7

T1 refers to the first data collection time point, T2 refers to the second data collection time point, and T3 refers to the third data collection time point. T1, T2, and T3 are subject-specific. Starting Quarter refers to the quarter in which participants were enrolled into the study. Any data during COVID-19 refers to whether participants provided at least one follow-up data point during the COVID-19 pandemic

**Table 2:** Correlation matrix of study variables

Variables	1	2	3	4	5
1. T1 loneliness	1				
2. T2 loneliness	0.862***	1			
3. T3 loneliness	0.812***	0.891***	1		
4. Parent RQ	− 0.531***	− 0.656***	− 0.653***	1	
5. Friend RQ	− 0.659***	− 0.472**	− 0.487**	0.269**	1

*p < 0.05, **p < 0.01, ***p < 0.001

### Baseline Analyses

Multiple linear regression analysis showed negative associations between both parent and friend relationship quality and loneliness at baseline (parent: *b* = − 0.28, SE = 0.05, t(97) = − 5.40, *p* < 0.001; friend: *b* = − 0.50, SE = 0.06, t(97) = − 8.10, *p* < 0.001). Post-hoc analyses using the single-item question about relying on older or new friends revealed that relying on friends established prior to college(or, alternately, relying on new friends) did not moderate the effect of friend relationship quality on loneliness (*b* = 0.07, SE = 0.12, *t*(95) = 0.59, *p*-value = 0.55). Additionally, results showed no significant interaction between parent and friend relationship quality (*b* = 0.004, SE = 0.087, t(96) = 0.047, *p* = 0.96) meaning that the effect of parent relationship quality on loneliness was not dependent on friend relationship quality.

Results from a semi-partial correlation showed that friend relationship quality accounted for more variance in loneliness compared to parent relationship quality: relationship quality with friends uniquely accounted for 29.56% of the variance in self-reported loneliness after partialling out the effect of parent relationship quality, whereas parent relationship quality uniquely accounted for 13.84% of the variance in self-reported loneliness.

### Growth Curve Modeling

**Fig. 2:**
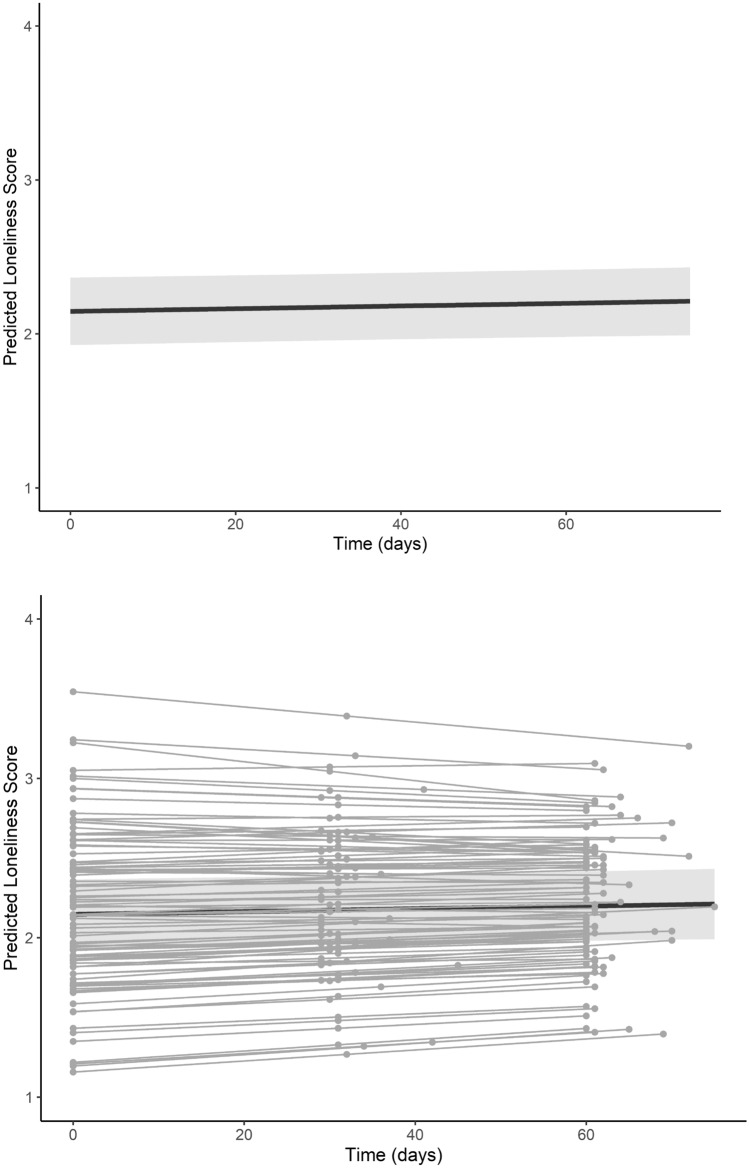
Longitudinal trajectories of loneliness. Note: Time is centered on the date of each participant’s initial assessment. The top panel shows the fixed effect of the relationship between time and loneliness. The bottom panel superimposes the random effects of each subject on the fixed effect curve. A 95% confidence interval is depicted

**Fig. 3:**
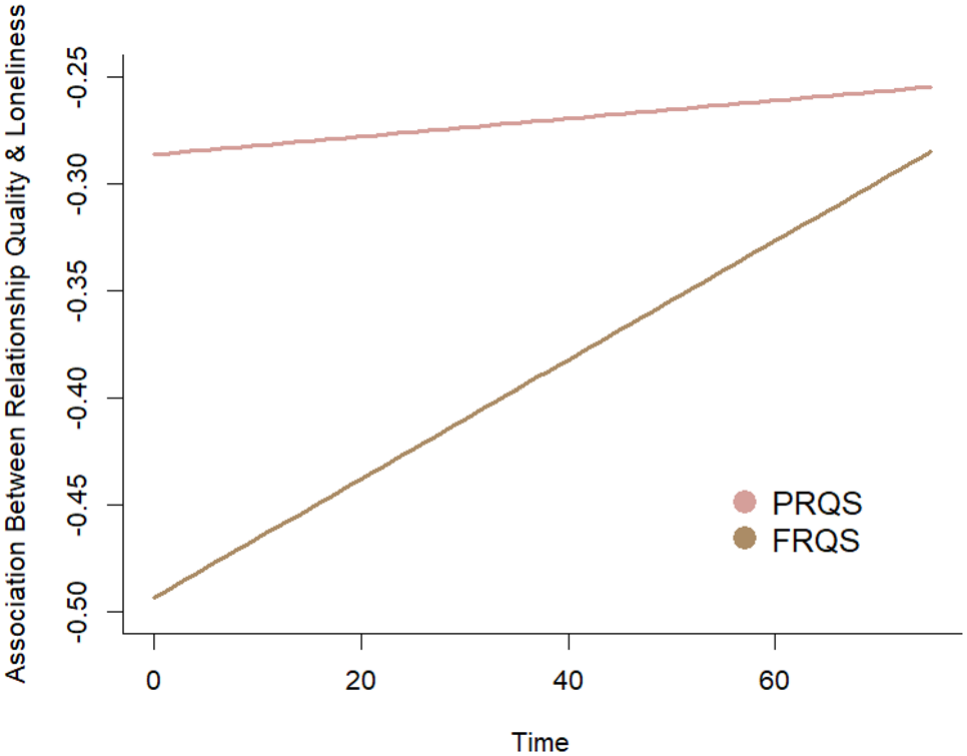
Loneliness trajectories as a function of parent and friend relationship quality. Note: PRQS refers to parent relationship quality score; FRQS refers to friend relationship quality score. Linear Growth Curve refers to the expected trajectory of loneliness over time conditional on a given level of parent or friend relationship quality. Notably, this plot indicates how the association between loneliness and relationship quality changes as a function of baseline, self-reported parent and friend relationship quality

Results from the modeling procedure are listed in Table 3. After partialling out the effects of between-person covariates (e.g., sex, quarter start, any COVID-19)—none of which were statistically significant—there were significant main effects of parent and friend relationship quality on loneliness as well as a trend towards increased loneliness over time (Fig. 2). Step 3 of our modeling procedure showed a significant interaction between friend relationship quality, such that the association between friend relationship quality and loneliness was attenuated over time (Fig. 3). By contrast, there was no such interaction between parent relationship quality and time, suggesting that association between parent relationship quality and loneliness remained constant over time (Fig. 3). These results highlight that trajectories of loneliness during the first year of college are conditional upon levels of relationship quality, and tend to increase overall. Supplementary results showed these interactions were preserved when also controlling for race, ethnicity, and first-generation college student status. These analyses also showed a main effect of race such that non-white participants were lonelier across the study than white participants, but did not exhibit differences in trajectories of loneliness (i.e., there was no interaction between time and race). There were also no interactions between time and first-generation student status.

**Table 3:** Growth curve modelling results

Predictor	Step 1	Step 2	Step 3
Intercept	2.186 (0.040)***	2.090 (0.112)***	2.091 (0.111)***
Time	0.001 (0.001)	0.001 (0.001)	0.001 (0.001)’
Parent RQ	− 0.269 (0.050)***	− 0.263 (0.051)***	− 0.277 (0.054)***
Friend RQ	− 0.416 (0.061)***	− 0.413 (0.064)***	− 0.486 (0.067)***
Sex	–	0.133 (0.093)	0.134 (0.093)
Quarter start	–	− 0.005 (0.146)	− 0.005 (0.146)
Any COVID	–	− 0.009 (0.130)	0.008 (0.130)
Parent RQ × time	–	–	0.000 (0.001)
Friend RQ × time	–	–	0.003 (0.001)**
SD(*e*_*ti*_)	0.289	0.281	0.260
SD(π_0*i*_)	0.288	0.297	0.302
SD(π_1*i*_)	0.002	0.002	0.002
Cor(π_0*i*_, π_1*i*_)	− 0.024	− 0.025	0.268
AIC	183.929	195.883	212.287
BIC	216.895	239.713	263.323

‘*p* < 0.10, *p < 0.05, **p < 0.01, ***p < 0.001; RQ refers to relationship quality obtained via the IPPA self-report instrument, time was coded as days since baseline (zero = day of baseline assessment); Sex was dummy coded (0 = male, 1 = female); quarter start referred to the academic quarter that a given participant enrolled in (dummy coded 0 = fall, 1 = winter); any COVID referred to whether a participant provided any data during any point during the COVID-19 pandemic (dummy coded 0 = no data provided during COVID-19 pandemic, 1 = at least one follow-up data point collected during COVID-19 pandemic). SD refers to standard deviation of conditional random effects; Cor refers to correlations between conditional random effects; AIC/BIC refer to Akaike and Bayesian Information Criterion, respectively

## Discussion

Social relationships can be a powerful source of resilience during times of developmental transition. The transition period from adolescence to adulthood is one of the most critical yet understudied in the lifespan, carrying with it the potential to significantly influence downstream adjustment outcomes. The present study investigated how close relationships affect loneliness during the first year of college, an important ‘rite of passage’ for many of those transitioning out of adolescence. We found that multiple close relationships can buffer individuals against loneliness during the first year of college, a period characterized by tremendous change. As predicted, there was a negative association between both parent and friend relationship quality and loneliness at baseline. Contrary to our prediction, there was no significant interaction between parent and friend relationship quality, suggesting that the effects of multiple close relationships are additive (i.e., the effect of one variable is constant over all levels of the other). However, these buffering effects observed at baseline had different consequences for trajectories of loneliness. Individuals with high parent relationship quality enjoyed a sustained buffer against loneliness 1 month and 2 months after baseline. By contrast, the buffering effect of friend relationship quality on loneliness became attenuated over time. These results contribute to the literature highlighting the importance of close relationships during the transition from adolescence to adulthood, underscore the independent effects of high-quality parent and friend relationships on well-being, and are informative for the development of interventions.

### Independent Contributions of Parent and Friend Relationships on Loneliness

The present study provides further support for the importance of parents and friends during the first year of college and gives insight into how these relationships contribute to adjustment. Parent and friend relationship quality were negatively associated with loneliness and cumulatively accounted for 43.4% of the variance in baseline loneliness. Given growing concerns about mental health on college campuses [64], this signals close relationships as a contributor to well-being and potential target for interventions. Additionally, the effect of parent relationship quality on loneliness was not dependent on friend relationship quality which is meaningful because it indicates that having both high parent and friend relationship quality does not provide an emergent benefit (i.e., it is not greater than the sum of its part) but also that having low relationship quality with both does not confer additional detriment. Put differently, it means additional improvements in parent (or friend) relationship quality affect individuals the same regardless of whether they are already high or low in friend (or parent) relationship quality. This suggests interventions targeting either parent or friend relationships might be beneficial for improving psychological well-being in college students. In particular, interventions aimed at promoting connectedness and relatedness—either through peer support programs, campus initiatives, or parent involvement—may be particularly valuable. Concretely, such interventions could entail brief, single-session treatments that are logistically favorable—such as parenting workshops during college move-in on how to stay connected with one’s children while supporting their autonomy—or peer counseling programs to help create structured opportunities to establish novel friendships and develop support systems.

These results indirectly suggest that parents and friends play different roles in a young adult’s life. Parent–child relationships remain relatively stable throughout college, and having higher parental support during the first year of college is associated with lower loneliness and higher academic persistence [65]. Parents are seen as a reliable source of guidance as young adults further individualize [66, 67]. As young adults transition to college, parents are a source of financial support and life advice [68] whereas friends are important for identity formation and fostering autonomy [69]. Interestingly, although young adults rate their parents as the most important people in their lives and refer to their parents for advice on important life decisions, on a day to day basis they seek out their friends more to discuss problems and consult their friends on topics such as dating, academic stressors, and college life [48]. When young adults do seek out their parents in making a decision, they seek an additional perspective on the decision rather than a definitive answer of what decision to make [70].

Friend relationship quality accounted for more variation in baseline loneliness (29.56%) compared to parent relationship quality (13.84%) suggesting that friends may be more adept than parents at fulfilling social needs during the initial transition to college. This is consistent with work by Dennis et al. [71], which found that peer support was a stronger predictor of college adjustment than family support. First-year college students exhibit psychological and behavioral phenotypes similar to those of adolescents [4] which together with the social, academic and geographic transitions associated with college might partly explain why they are sensitive to friendship disruptions [12]. Relationships with friends in young adulthood are instrumental for identity exploration, meeting social needs and, for new college students, might serve as a marker of integration to college [40, 69].

Importantly, the present study found that relationship quality with parents and friends not only differentially impacted loneliness at baseline but also longitudinally, reflecting the importance of quality close relationships for overall well-being during young adulthood [72]. While parent relationship quality continued to predict less loneliness over time, friend relationship quality decreased in predictive power (though it is worth noting the apparent buffer of friend relationship quality on loneliness did not disappear completely). It is possible that over time the contextual factors that influence loneliness during the first year of college outweigh the benefits of having high friend relationship quality. The diminished relationship between friend relationship quality and loneliness over time might reflect a growing tendency to interact with friends in less satisfying ways [73]—for example, in virtual formats due to increasing academic demands—or changes in friend groups and subsequent fluctuations in social support. In support of this latter possibility, prior research in young adults suggests that network composition turnover can be as high as 40% in 1 year [74]. That parent relationship quality’s effects on loneliness did not depend on time suggests that parents may serve as pillars of stability in periods of time where individuals experience friendship instability, though more work is needed to directly verify this possibility.

### Trajectories of Loneliness

We also sought to characterize typical loneliness trajectories in the first year of college. Relative to prior samples of college students, our sample had similar levels of loneliness at baseline [30, 31, 75, 76]. Results showed that, after adjusting for covariates, overall loneliness tended to increase over time albeit modestly. It is worth noting that the effect was present across participants regardless of whether they enrolled in the study during the Fall or Winter quarter of their first year of college. It is possible that after the initial novelty and excitement of entering college has subsided, individuals are faced with unmet expectations about college, friendship instability, and the day to day stressors of being a college student [12, 19, 21]. Although students often form friendships at the start of college, these relationships are likely not as established as those they had before college, which may leave them without a reliable support network [12]. At the same time, relying on high school friends beyond the first few weeks of college can hinder adjustment [40], and relationship satisfaction with close high school friends typically declines across the first year of college [50].

Remarkably, the COVID-19 pandemic did not impact loneliness ratings nor did it impact how relationship quality moderated loneliness. This points to two things: (1) that loneliness is a highly subjective experience (even in the face of mandatory social distancing), and (2) that relationship quality can buffer individuals against loneliness even in fairly extreme situations. Although the present study did not find an impact of the COVID-19 pandemic on loneliness, other work found elevated loneliness in college students and young adults more broadly during the pandemic [38, 77]. Students experienced many COVID-19 related stressors such as the abrupt transition to online instruction, changes in living situation, concerns of infection, and financial difficulties which likely exacerbated existing psychological distress [39, 78]. Another study in college students found that greater social support during the pandemic was associated with less stress and greater happiness reiterating the value of high quality relationships [79].

The results of the present study are a departure from prior literature, which has found evidence for decreasing loneliness from the beginning to the end of the first year of college. Cutrona [32] and Shaver et al. [12] found trends of decreases in loneliness from Fall quarter to Spring quarter of the first year of college. Our findings speak to possible changes in the mental health of current cohorts of college students compared to students from 40 years ago and even just 10 years ago. Emotional well-being of first-year college students has been on a steady decline since 1985, with only 50.4% of men and 34% of women reporting above average emotional well-being in 2019 compared to 68.1% of men and 59.3% of women in 1985 [80, 81]. Additionally, in 2019, 16.6% of first-year college students reported frequently feeling depressed whereas in 2009 the rate was 6.1% [81]. The present trend in loneliness in college students may also speak to generational differences in loneliness during young adulthood within the context of an ever-changing society. A study by Cigna in 2018 found that Generation Z, defined in the study as adults 18–22 (born 1996–2000), is the loneliest generation, with 68% reporting that they feel that no one really knows them well. Given these societal shifts in loneliness trends, it is important to reassess loneliness during young adulthood with each generation and not assume that past trends still apply. Alternately, it is possible that differences between our results and older literature stem from analytic differences given our use of multiple timepoints and growth curve models [62, 82].

Overall, however, it is necessary to note that trajectories of loneliness were ultimately conditional on levels of friend relationship quality. This reinforces the notion that a ‘one-size-fits-all’ characterization of the first year of college does not apply to all students—rather, different students go through different experiences that engender different outcomes. This emphasizes a stronger need to perhaps focus on individual differences when studying developmental transitions, especially ones that are structural and social in nature.

### Social Relationships as a Potential Intervention Target

Loneliness inflicts a toll on well-being and has negative downstream consequences, therefore, knowing the effect of parent and friend relationship quality on loneliness can inform interventions. The results of the present study indicate that targeting relationship quality, especially friend relationship quality, could be effective in mitigating loneliness during the first year of college. Interventions for students that are struggling with elevated loneliness could incorporate strategies that improve relationship quality with parents and friends as a means to reduce loneliness. For example, parent relationship quality could be improved by providing more opportunities for first-year college students to interact with their parents (e.g., hosting more parent visiting events) and encouraging consistent communication between them. Prior research found that high-quality interactions with parents during the first year of college predicted greater positive affect and lower negative affect for that particular day [83]. Meanwhile, overall friend relationship quality could be targeted through college events that cultivate greater community among first-year students and provide opportunities to meet new people and bond with existing friends. Bohnert et al. [84] found that participation in community activities fostered greater friend relationship quality in individuals who had poor friend relationships before college. Therefore, encouraging involvement in campus events could be effective in targeting new friend relationships. Further, our supplementary findings showing that non-white individuals were more likely to be lonelier than their white counterparts imply further intervention specificity. These findings carry importance outside the scope of the first year of college, as they also imply that parent and friend relationship quality can potentially help buttress individuals during other seminal moments early on in adulthood, such as entering the workforce, getting married, or having children. While we have no data to speak directly to this point, it is a noteworthy consideration for future research.

### Limitations and Future Directions

The present study has several limitations including the sole use of self-report measures. Due to the nature of the IPPA Peer survey, we were unable to determine if relationships with new college friends primarily accounted for friend relationship quality scores. This could be addressed in future work by having participants complete the IPPA Peer measure with only their college friends in mind or asking them to specify what friends they were thinking of when completing the questionnaire. Additionally, using the IPPA as our measure of relationship quality provided an overview of parental and friend relationship quality but it did not provide insight into what aspects of the relationship made it high quality (e.g. responsiveness, level of trust, communication) as well as what feature of high-quality relationships is most influential to loneliness outcomes. Given that the study sample was largely female, it also was not possible to delve into the role of sex on perceived parental relationship quality as well as the interaction between participant sex and sex of parent. Furthermore, the present study assessed trajectories of loneliness during a part of the first year of college, not the entirety of the first year. Future studies would benefit from continuously assessing loneliness over the entire course of the first year of college (i.e., from orientation to the end of the first year of college) in order to more comprehensively characterize the trajectory of loneliness across the first year of college.

Future studies might aim to further examine the association between friend relationship quality and loneliness to determine what aspects of friend relationships are particularly helpful in mitigating loneliness. Future work should also assess whether outcomes like depression and anxiety are sensitive to the influence of relationship quality with parents and friends. Prior work has found that poorer family relationship quality predicts onset of depression in young adults [85]. Importantly, having a high quality relationship with parents ideally entails an appropriate degree of independence, given prior work linking “failure to launch” from the home environment with anxiety in emerging adults [86]. Given the prevalence of anxiety and depression among college populations [64], it would be beneficial to assess whether parent and friend relationship quality could strongly buffer against those psychological outcomes.

A final consideration involves generalizability to other contexts during the transition from adolescence to young adulthood. It may be inappropriate to assume the relationships observed here extrapolate to other contexts (e.g., end of college, individuals attending 2 year programs, trade school, entering the workforce, etc.). While it is a worthwhile point for future research to determine whether the ostensible buffering effects observed here are present in other contexts, we remind readers that it may be inappropriate to extrapolate from our current findings without additional work.

### Summary

The beginning of college often involves both challenges and opportunities as one adjusts to a new academic and social environment. Individuals may be particularly vulnerable to loneliness during this transition, putting them at risk for both psychological and academic difficulties. The present longitudinal study characterized typical trajectories of loneliness and examined whether high-quality relationships with parents and friends could be a potential buffer against loneliness during the first year of college. The study sample consisted of ethnically diverse first-year college student at a large public university in the Western United States. Relationship quality measures were obtained at baseline and loneliness was assessed at 3 time points (baseline, 1 month and 2 months later). We found that individuals with high relationship quality with parents and friends had lower loneliness at baseline, and the relationship between parent relationship quality and loneliness stayed constant over time whereas the effect of friend relationship quality was attenuated. We also found that loneliness increased over time, which is in contrast to older work that found a decrease in loneliness from the beginning to the end of the first year of college. These results emphasize the importance of high quality relationships with both parents and friends and suggest the potential for interventions aimed at strengthening said relationships. Thus, in future work, relationship quality with close others could be targeted to mitigate loneliness which could, in turn, lessen the risk of the negative outcomes associated with loneliness.

### Supplementary Information

Below is the link to the electronic supplementary material.

Supplementary file1 (DOCX 32 kb)

## Footnotes

1 This model also estimates ⍴, the first-order autoregressive parameter helps determines covariances between level-1 error at two timepoints pursuant to the form σ^2^(⍴^h^), where σ^2^ is the level-1 error variance and *h* is the lag between timepoints (see Fujikoshi et al. [63]).
