# Supplementary material for: Parent and Friend Relationship Quality and Links to Trajectories of Loneliness During the First Year of College
Source: Child Psychiatry Hum Dev. 2022 Sep 24;55(3):680–94. doi: 10.1007/s10578-022-01416-6 (PMC9510327; doi:10.1007/s10578-022-01416-6)
Supplement: Supplementary file 1 — Supplementary file1 (DOCX 32 kb) [file 10578_2022_1416_MOESM1_ESM.docx]

**Supplement**

Parent and Friend Relationship Quality and Links to Trajectories of Loneliness during the First Year of College

**Other Measures**

*Self-report surveys*

*Academic and Non-academic Activities

*Screen for Adult Anxiety Related Disorders Questionnaire (SAARED - AdultVersion)

*Risk Perception Measure (RPM)

*Brief Sensation Seeking Scale (BSSS)

*Center for Epidemiologic Studies Depression Scale (CESD)

*Childhood Trauma Questionnaire –Short Form (CTQ)

*Activities Questionnaire

*Pittsburgh Sleep Quality Index (PSQI)

*Domain-Specific Risk-Taking Adolescent’s Scale (DOSPERT-adolescent)

*Expanded Emotion Regulation Questionnaire (E-ERQ)

*Frost Multidimensional Perfectionism Scale (FMPS)

*Implicit Beliefs of Emotion Scale

*Launching Emerging Adults Form (LEAF)

*Patient Health Questionnaire (PHQ-9)

*Rosenberg Self-Esteem Scale

*Social Interaction Anxiety Scale (SIAS)

*Social Networks Questionnaire

*Social Support

*Theories of Intelligence

*Undergraduate Stress Questionnaire

*Ambiguity Tolerance (MSTAT-II)

*Computerized tasks*

*Risky Decision-Making Task (Sokol-Hessner et al., 2009; Sokol-Hessner et al., 2015)

*Vanishing Bandit Task (Navarro et al., 2018)

*Risk Ambiguity Task (Levy et al., 2010; Tymula et al., 2012)

*Supplementary Table 1.* Descriptive statistics for additional demographic data

| Variables | N | M (SD) |
| --- | --- | --- |
| Race  White  Non-White | 27  70 |  |
| Ethnicity  Latinx  Not Latinx | 24  77 |  |
| First Generation  Yes 38   No 63 | | |

*Note.* The ‘Race’ variable reported here categorized students who indicated Caucasian as their only race as ‘White’ and those who indicated any other race, or mix of races, as ‘Non-White’. We note that this is not an optimal categorization, but note that several races that we assessed had very little participants and it would have been statistically inadvisable to use a set of dummy or effects coded categorical predictors (e.g. grossly imbalance, to few cases to reliably estimate a coefficient for a group, etc.). ‘Ethnicity’ referred to whether individuals identified as Hispanic/Latinx or not. Notably, these are distinct concepts (one can be racially white and Latinx/Hispanic, or racially Black and Latinx/Hispanic). ‘First Generation’ refers to whether individuals are the first person in their immediate family to attend college.

*Supplementary Table 2.* Growth curve modeling results without imposing AR(1) structure.

| Predictor | Step 1 | Step 2 | Step 3 |
| --- | --- | --- | --- |
| Intercept | 2.195 (0.039)*** | 2.096 (0.113)*** | 2.096 (0.113)*** |
| Time | 0.001 (0.001) | 0.001 (0.001) | 0.001 (0.001)’ |
| Parent RQ | -0.271 (0.051)*** | -0.264 (0.052)*** | -0.274 (0.054)*** |
| Friend RQ | -0.424 (0.062)*** | -0.419 (0.065)*** | -0.473 (0.067)*** |
| Sex | - | 0.141 (0.094) | 0.140 (0.094) |
| Quarter Start | - | -0.013 (0.148) | -0.008 (0.148) |
| Any COVID | - | -0.004 (0.131) | -0.008 (0.131) |
| Parent RQ x Time | - | - | 0.000 (0.001) |
| Friend RQ x Time | - | - | 0.003 (0.001)** |
| SD(*e_ti_*) | 0.183 | 0.183 | 0.183 |
| SD(π_0_*_i_*) | 0.357 | 0.358 | 0.356 |
| SD(π_1_*_i_*) | 0.004 | 0.004 | 0.003 |
| Cor(π_0_*_i_*, π_1_*_i_*) | -0.077 | -0.075 | -0.030 |
| AIC | 184.339 | 196.027 | 213.550 |
| BIC | 213.643 | 236.205 | 260.941 |
| *Note:* ‘*p*<.10 **p<.05 ** p< .01 *** p<.001;* RQ refers to relationship quality obtained via the IPPA self-report instrument, time was coded as days since baseline (zero = day of baseline assessment); Sex was dummy coded (0 = male, 1 = female); quarter start referred to the academic quarter that a given participant enrolled in (dummy coded 0 = fall, 1 = winter); Any COVID referred to whether a participant provided any follow-up data during any point during the COVID-19 pandemic (dummy coded 0 = no data provided during COVID-19 pandemic, 1 = at least one data point collected during COVID-19 pandemic). SD refers to standard deviation of conditional random effects; Cor refers to correlations between conditional random effects; AIC/BIC refer to Akaike and Bayesian Information Criterion, respectively. | | | |

*Supplementary Table 2.* Growth curve modeling results with additional demographic variables

| Predictor | Step 1 | Step 2 | Step 3 |
| --- | --- | --- | --- |
| Intercept | 2.178 (0.042)*** | 1.898 (0.127)*** | 1.923 (0.131)*** |
| Time | 0.001 (0.001) | 0.001 (0.001) | 0.001 (0.001) |
| Parent RQ | -0.277 (0.051)*** | -0.242 (0.051)*** | -0.259 (0.056)*** |
| Friend RQ | -0.415 (0.062)*** | -0.415 (0.063)*** | -0.497 (0.069)*** |
| Sex | - | 0.165 (0.095)’ | 0.166 (0.095) |
| Quarter Start | - | -0.056 (0.149) | -0.051 (0.149) |
| Any COVID | - | -0.032 (0.134) | -0.036 (0.134) |
| First Gen | - | 0.084 (0.094) | 0.082 (0.094) |
| Ethnicity | - | 0.030 (0.110) | 0.030 (0.111) |
| Race | - | 0.244 (0.088)** | 0.208 (0.096)* |
| Race x Time | - | - | 0.001 (0.001) |
| Parent RQ x Time | - | - | 0.001 (0.001) |
| Friend RQ x Time | - | - | 0.003 (0.001)** |
| SD(*e_ti_*) | 0.333 | 0.318 | 0.285 |
| SD(π_0_*_i_*) | 0.249 | 0.250 | 0.284 |
| SD(π_1_*_i_*) | 0.000 | 0.000 | 0.000 |
| Cor(π_0_*_i_*, π_1_*_i_*) | -0.005 | -0.005 | -0.003 |
| AIC | 179.000 | 195.669 | 226.064 |
| BIC | 211.681 | 249.811 | 290.836 |
| *Note:* ‘*p*<.10 **p<.05 ** p< .01 *** p<.001;* RQ refers to relationship quality obtained via the IPPA self-report instrument, time was coded as days since baseline (zero = day of baseline assessment); Sex was dummy coded (0 = male, 1 = female); quarter start referred to the academic quarter that a given participant enrolled in (dummy coded 0 = fall, 1 = winter); Any COVID referred to whether a participant provided any follow-up data during any point during the COVID-19 pandemic (dummy coded 0 = no data provided during COVID-19 pandemic, 1 = at least one data point collected during COVID-19 pandemic); First Gen refers to first generation college student status, dummy coded 0 = no, 1 = yes; Race was dummy coded 1 = Non-White, 0 = White; Ethnicity was dummy coded 1 = Latinx/Hispanic, 0 = Not Hispanic/Latinx. *N* subjects = 97, *n* datapoints = 283. SD refers to standard deviation of conditional random effects; Cor refers to correlations between conditional random effects; AIC/BIC refer to Akaike and Bayesian Information Criterion, respectively. An AR(1) structure was imposed on the level 1 error covariance matrix for these models. | | | |
